# Correction: Knowledge of COVID-19 and the impact on indigents’ access to healthcare in Burkina Faso

**DOI:** 10.1186/s12939-022-01797-z

**Published:** 2023-01-12

**Authors:** E. Bonnet, Y. Beaugé, M. F. Ba, S. Sidibé, M. De Allegri, V. Ridde

**Affiliations:** 1 Institut de Recherche Pour Le Développement, UMR 215 PRODIG, 5, Cours Des Humanités, 93 322 Aubervilliers Cedex, France; 2 Heidelberg University, University Hospital and Medical Faculty, Heidelberg, Germany; 3 Institut de Santé Et de Développement (ISED), Cheikh Anta Diop University, Dakar, Senegal; 4 University Joseph Ki-Zerbo of Ouagadougou, Ouagadougou, Burkina Faso; 5 Institut de Recherche Pour Le Développement, Ceped, Université de Paris, Inserm ERL 1244, 45 Rue Des Saints-Pères, 75006 Paris, France; 6 Institut de Santé Et Développement, Université Cheikh Anta Diop, Dakar, Senegal

**Correction: Int J Equity Health 21, 150 (2022)**

**https://doi.org/10.1186/s12939-022-01778-2**

After publication of this article [1], the authors reported that in the first sentence of the Methods-section, “(GODIN)” should have read “[30]”.

Reference [30] has been added, as follows:

Godin G, Kok G. The theory of planned behavior: a review of its applications to health-related behaviors. Am J Health Promotion. 1996;11(2):87-98. doi:10.4278/0890-1171-11.2.87.

The original article [1] has been corrected.
